# Poor linkage to care for HIV-positive OVC with disabled caregivers: a longitudinal study in Tanzania

**DOI:** 10.1186/s12889-021-10415-6

**Published:** 2021-02-16

**Authors:** Shraddha Bajaria, Amon Exavery, Noreen Toroka, Ramadhani Abdul

**Affiliations:** 1 Ifakara Health Institute, Dar es Salaam, Tanzania; 2 Pact, Dar es Salaam, Tanzania

**Keywords:** OVC, HIV, Disability, Linkage to care

## Abstract

**Background:**

Despite extensive efforts to scale up counseling and testing services and care and treatment clinics (CTCs) in Tanzania, linkage between points of diagnosis and CTCs remains low. Studies have looked at barriers such as lack of trained health providers, poor referral system, economic costs or distance to health facilities, but fewer assessed the association between caregivers’ vulnerability such as disability and linkage of orphans and vulnerable children (OVCs) in their care to health facilities. This study describes the magnitude of caregivers’ disability and assesses its relationship with successful linkage to care of their OVC living with HIV/AIDS in Tanzania.

**Methods:**

Data for this analysis came from the USAID Kizazi Kipya project in 79 councils of Tanzania. Data on HIV risk, service use and ART adherence among OVC aged 0–19 years were collected during the project’s quarterly routine data collection (Oct 2017-Sep 2018). Characteristics of caregivers were collected during the project beneficiary screening and enrollment process. Generalized estimating equation models were used to analyze the factors that are associated with linkage of 14,538 HIV positive OVC to CTC, who were taken care of by 11,834 caregivers.

**Results:**

The majority of caregivers (70%) were females, had completed primary education (67%), 54% were married or cohabiting. Of all the OVC, 3% were living with disabled caregivers; of whom 89% were physically disabled while 11% were mentally disabled. OVCs living with disabled caregivers were less likely to be linked to care (OR 0.76, 95% CI 0.58, 0.99). Factors positively associated with OVC linkage to care were high caregivers’ education level (OR 1.99, 95% CI 1.51, 2.63) and OVC living with a HIV positive caregivers (OR 1.25, 95% CI 1.12, 1.41). OVC living in household with high socio-economic status were less likely to be linked to care (OR 0.76, 95% CI 0.67, 0.86) than those in low-SES households.

**Conclusion:**

These results suggest HIV positive OVC living with disabled caregivers had poor linkage to care. The findings highlighted the need to focus attention to the disabilities-led household to promote inclusion and improve access to the HIV services.

## Background

HIV/AIDS remains a major public health threat in sub-Saharan Africa, disproportionately carrying more than two thirds of the global burden of the infection [1] and home to the largest number of people living with HIV (PLHIV) [1]. In 2018, UNAIDS estimated that 1.6 million people were living with HIV in Tanzania; of which 78% were aware of their status, of which 92% were receiving treatment, and 87% of those on treatment were virally suppressed [2]; similar results were reported in the National AIDS Control Programme guideline in 2019 [3]. In efforts of achieving the UNAIDS 90–90-90 goals, approaches such as voluntary counselling and testing, home-based and community testing have been introduced to reach all PLHIV and care and treatment clinics (CTCs) have been widely set up to provide timely ART to PLHIV in Tanzania in the last decade [4].

There is little literature available on the overall linkage to care in Tanzania, and none to the authors’ knowledge for OVC of disabled caregivers. According to the Tanzanian HIV testing and counseling guidelines [5], all HIV positive individuals should be linked to care and treatment services [5, 6]. Additionally, all HIV testing sites are required to establish referrals to CTCs [5]. Despite of these large-scale efforts, linkages between points of diagnosis and enrollment to CTCs remain reportedly poor [4, 7, 8]. Few studies from different regions of the country have reported low linkage to care; 14% in Mwanza [7] and 23.1% in Ifakara [8]. Barriers to linkage at community, facility, and individual levels of the continuum of care are studied widely; most commonly reported are weak referral systems from testing to treatment sites, unpleasant experience at a health facility, lack of human resource at facility, distance to health facilities, undisclosed HIV status, stigma and fear, reluctance to receive HIV services [4, 7–9].

In 2016, 8% of the children under 18 years of age were orphans in Tanzania [10]; 31% of these children were orphans due to AIDS [10, 11]. Orphanhood due to HIV increases risk of HIV infection as well as other negative health and social outcomes for children [12, 13]. OVC are often cared for by a living parent, grandparents or other family relatives and are dependent on them for basic needs [14], who might be old in age, ill or disabled. Some studies have shown the negative consequences faced by OVC due to economic or social constraints of their caregivers, such as low school enrollment, nutritional deficiencies, inadequate shelter, lack of psychosocial support, adverse health outcomes [6, 15]. Orphans living with HIV (OVCLHIV) often have delayed access to care and treatment [16]. The consequences can be even poorer if the caregiver is disabled, due to the high dependence of children on caregivers.

In Tanzania, about 5.9% of the population was disabled in 2012 [17]. The highest reported disabilities were impairments in sight, walking and remembering [17, 18]. The socio-economic challenges faced by disabled household heads are often reported; there is an increased economic vulnerability due to challenges in accessing education [18, 19] as well as higher dependency on self-employment and agriculture due to employment discrimination, employer ignorance and lack of awareness, despite the existing Persons with Disabilities Act, requiring all employers of a workforce of twenty or more people to hire at least 3% employees with disabilities [20]. Disability-associated stigma is also common [21], leading to inaccessibility of health services and poor health outcomes, for both, the caregivers and the children they take care of. Awareness of HIV/AIDS spread and prevention is also low among the disabled, due to very few programs, policies and campaigns centering the needs of disabled people [18, 22]. This in turn makes the disabled population at high risk of HIV and adverse HIV related outcomes [22]. The lack of knowledge about HIV and inaccessibility to health services could also make it difficult for disabled caregivers to look after their OVCLHIV and accompany them to the health facilities to access treatment.

While the barriers and challenges faced by the disabled population in accessing HIV services have been reported [18, 22, 23], there remains a gap in literature whether disability of caregivers of HIV positive children hinders access to care and treatment of HIV. This study describes the magnitude of caregivers’ disability and assesses its relationship with successful linkage to care of their OVC living with HIV/AIDS in Tanzania. Specifically, this study explores the socio-demographic characteristics of OVCLHIV who were linked to care and compares linkage of the OVCLHIV to care between those living with a disabled caregivers versus not.

## Methods

### Study design, site, and population

This secondary analysis used existing data from the USAID Kizazi Kipya project. The project provides health, social and economic strengthening services to eligible OVC and their caregivers via community cadres from Civil Society Organizations (CSOs) by providing bi-directional referrals at national, regional and district levels. This analysis includes data from the 79 councils where activities were implemented in the second project year (October 2017 to September 2018). Caregivers of all HIV positive OVC (11,834 caregivers of 14,538 HIV positive OVC) within the study period were included in this analysis.

### Study tools

After eligible OVC and their caregivers are enrolled into the project, the project’s Family and Child Asset Assessment (FCAA) tool is administered, to evaluate the household’s vulnerabilities, needs and strengths. OVC, caregivers and household information is collected and provides a basis on which a household care plan should be developed. Data on OVC’s HIV risk assessment and adherence is collected twice a year using the project’s data collection tool. The aim of this tool is to understand if the OVC is at risk for HIV infection, if an HIV positive OVC has been enrolled to CTC or started ART or needs adherence support.

### Data source

Using the project’s standard data collection tools such as the FCAA and HIV risk assessment forms, data are routinely collected by community workers (CW) who are trained to support implementation of the project activities by visiting beneficiaries on a monthly basis and following up on the referrals and services needed or provided. These data are then electronically entered into the project’s data system.

### Details of measurement

For the project, linkage to care is recorded in the HIV risk assessment data. OVCLHIV or their caregivers are asked whether the OVC has been receiving services at the CTC. If the response is yes, CTC-1 card is asked for verification. For this study, linkage to care is defined as ‘yes’ if the caregiver reported ‘yes’ in the HIV risk assessment, and ‘no’ if otherwise. Information on whether the caregiver has any disability is self-reported during the FCAA. If yes, the caregivers also report the type (physical or mental) of disability.

Principal component analysis (PCA) was used to create socio-economic status (SES) levels for the households, for which three tertiles were created: low, medium and high, using the household assets reported to be owned during the FCAA. Household assets such as the dwelling material, livestock owned (chicken, goats, cows), transport vehicles owned (bicycle, motorbike, tractor, rickshaw), sewing machine, television, radio, sofa, hair dryer and kitchen appliances (oven, refrigerator, blender, freezer, cooking gas) owned, were used to create the tertiles for PCA.

### Data management and analysis

Data were analyzed using Stata 15 software, using beneficiary (OVCLHIV) as the unit of analysis. To take into account the multi-level, hierarchical nature of the project’s data and the clustering effect of OVC within a household, and a household within a CW, generalized estimating equations (GEE) with logit link function utilizing a binomial distribution family and an exchangeable correlation structure were used to analyze the caregivers and household characteristics that are associated with OVC linkage to care.

## Results

### Characteristics of OVC

Of the 14,538 OVCLHIV analyzed, 7532 (51.8%) were female. Most (30.7%) OVC were between the age of 11 to 15 years, 27.8% were between 6 to 10 years old, 22% were between 0 to 5 years, 19.5% were above the age of 15 years. Of the 9880 OVC aged 8 years or above, 84% (8261) knew their HIV status, 14% (1354) had an undisclosed HIV status while 265 OVC had a missing disclosure status. Most of the OVCLHIV (*n* = 12,653, 87%) reported to be linked to care. Of the 1885 OVCLHIV who were not linked to care, 73 (4%) had disabled caregivers. More than half (*n* = 1051, 56%) of OVCLHIV who were not linked to care were provided with a referral; only 34 of the 73 OVC with a disabled caregiver were referred.

### Characteristics of caregivers of HIV positive OVC by caregiver disability status

Table 1 shows the socio-demographic characteristics of caregivers of all HIV positive OVC included in this study by their disability status. Overall, majority (70.9%) of the caregivers were females, with slightly higher males with a disability than females. Most caregivers were between the ages 26–40 (31.9%) and 41–50 (30.4%) years; disability was higher in older caregivers (p = 0.000). Most caregivers without a disability had completed primary education (67.3%); higher proportion of caregivers with a disability had never attended school (27.6%) compared to those without a disability (18.3%), *p* = 0.000. Half of the caregivers with a disability had never been married, 39.6% were divorced, separated or widowed; only 9.1% were married or cohabiting compared to 53.7% of caregivers without a disability. Almost two thirds (61.2%) of all caregivers reported to be HIV positive; significantly more caregivers without a disability were HIV-positive (61.9%), compared to those with a disability (35.8%).

**Table 1:** Characteristics of all caregivers of HIV positive OVC by caregiver disability status

	Caregivers without disability (***n*** = 11,493)		Caregivers with disability (***n*** = 341)		Total (n = 11,834)		***p***-value
	n	(%)	n	(%)	n	(%)	
**Sex**							0.002
Male	3315	(28.8)	125	(36.7)	3440	(29.1)	
Female	8178	(71.2)	216	(63.3)	8394	(70.9)	
**Age (in years)**							0.000
0–25	388	(3.4)	8	(2.4)	396	(3.4)	
26–40	3705	(32.2)	68	(19.9)	3773	(31.9)	
41–50	3506	(30.5)	93	(27.3)	3599	(30.4)	
51–60	1882	(16.4)	60	(17.6)	1942	(16.4)	
Above 60	2012	(17/5)	112	(32.8)	2124	(17.9)	
**Education level**							0.000
Never attended	2097	(18.3)	94	(27.6)	2191	(18.5)	
Primary incomplete	1122	(9.8)	52	(15.3)	1174	(9.9)	
Primary complete	7735	(67.3)	175	(51.3)	7910	(66.8)	
Post-primary	539	(4.7)	20	(5.9)	559	(4.7)	
**Marital status**							0.645
Never been married	908	(7.9)	31	(50.9)	939	(7.9)	
Married/cohabiting	6176	(53.7)	173	(9.1)	6349	(53.7)	
Divorced/separated/widowed	4363	(37.9)	135	(39.6)	4498	(38.0)	
Other	46	(0.4)	2	(0.6)	48	(0.4)	
**HIV status**							0.000
Positive	7115	(61.9)	122	(35.8)	7237	(61.2)	
Negative	2847	(24.8)	144	(42.2)	2991	(25.3)	
Undisclosed	1531	(13.3)	75	(21.9)	1606	(13.6)	
**Household socio-economic status**							0.105
Low	3700	(32.2)	123	(36.1)	3823	(32.3)	
Medium	3772	(32.8)	117	(34.3)	3889	(32.9)	
High	4021	(34.9)	101	(29.6)	4122	(34.8)	

### Characteristics of OVC by OVC linkage to care status

Table 2 presents the characteristics of OVC by the status of OVC’s linkage to care. Of all the OVC who were not linked to care, highest proportion were between the age of 11–15 years (28.9%), 30.9% of OVC linked to care were between the age of 11–15 years. Higher proportion of OVC linked to care were above 15 years (19.7%) compared to 17.9% of OVC who were not linked to care (p = 0.000). A higher proportion of OVC who had not been linked to care had caregivers who had never attended school (25.4%) compared to OVC who were linked to care (17.4%). Higher proportion of OVC linked to care had caregivers with a completed primary (67.9%) and post primary education (4.9%), *p* = 0.000. More OVC linked to care had HIV-positive (62.6%) caregivers compared to OVC not linked to care (53.3%). There was no significant difference in the type of disability of caregivers by OVC linkage to care.

**Table 2:** OVC characteristics by OVC linkage to care status

	OVC not linked to care (***n*** = 1885)		OVC linked to care (***n*** = 12,653)		***p***-value
	n	(%)	n	(%)	
**OVC characteristics**					
**Sex**					0.820
Male	913	(48.4)	6093	(48.2)	
Female	972	(51.6)	6560	(51.9)	
**Age (in years)**					0.000
0–5	512	(27.2)	2691	(21.3)	
6–10	490	(25.9)	3552	(28.1)	
11–15	546	(28.9)	3913	(30.9)	
Above 15	337	(17.9)	2497	(19.7)	
**Caregiver characteristics**					
**Sex**					0.777
Male	543	(28.8)	3685	(29.1)	
Female	1342	(71.2)	8968	(70.9)	
**Age (in years)**					0.082
0–25	63	(3.3)	409	(3.2)	
26–40	635	(33.7)	4037	(31.9)	
41–50	543	(28.8)	3969	(31.4)	
51–60	332	(17.6)	2033	(16.1)	
Above 60	312	(16.6)	2205	(17.4)	
**Education level**					0.000
Never attended	478	(25.4)	2203	(17.4)	
Primary incomplete	174	(9.2)	1237	(9.8)	
Primary complete	1168	(61.9)	8597	(67.9)	
Post-primary	65	(3.5)	616	(4.9)	
**Marital status**					0.000
Never been married	105	(5.6)	1057	(8.4)	
Married/cohabiting	1098	(58.3)	6680	(52.8)	
Divorced/separated/widowed	680	(36.1)	4866	(38.5)	
Other	2	(0.1)	50	(0.4)	
**HIV status**					0.000
Positive	1004	(53.3)	7925	(62.6)	
Negative	526	(27.9)	3105	(24.5)	
Undisclosed	355	(18.8)	1623	(12.8)	
**Household socio-economic status**					0.000
Low	533	(28.3)	4180	(33.0)	
Medium	608	(32.3)	4235	(33.5)	
High	744	(39.5)	4238	(33.5)	
**Disability (*n*** **= 420)**					0.941
Physical	65	(89.1)	310	(89.3)	
Mental	8	(10.9)	37	(10.7)	

Table 3 presents the results of univariable and multivariable GEE regression analyses of the factors associated with OVC’s linkage to care. OVC age was positively associated with linkage to care. Caregiver characteristics that were significantly associated with OVC’s linkage to care were disability, marital status, HIV status and education level.

**Table 3:** GEE logistic regression of factors associated with HIV positive OVC’s linkage to care

	Univariable (***n*** = 14,538)		Multivariable (***n*** = 14,538)	
	OR	(95% CI)	OR	(95% CI)
**Disabled Caregiver**				
No	1.0		1.0	
Yes	0.70**	(0.54, 0.91)	0.76*	(0.58, 0.99)
**Caregiver’s disability type**				
Mental	1.0			
Physical	1.03	(0.46, 2.32)		
**OVC sex**				
Male	1.0			
Female	1.01	(0.92, 1.11)		
**OVC age (in years)**				
0–5	1.0		1.0	
6–10	1.38***	(1.21, 1.58)	1.39***	(1.21, 1.59)
11–15	1.36***	(1.20, 1.55)	1.39***	(1.22, 1.59)
Above 15	1.41***	(1.22, 1.63)	1.45***	(1.24, 1.68)
**Caregiver sex**				
Male	1.0			
Female	0.98	(0.88, 1.10)		
**Caregiver age (in years)**				
0–25	1.0			
26–40	0.98	(0.74, 1.29)		
41–50	1.13	(0.85, 1.49)		
51–60	0.94	(0.71, 1.26)		
Above 60	1.09	(0.81, 1.46)		
**Caregiver marital status**				
Never been married	1.0		1.0	
Married/cohabiting	0.60***	(0.49, 0.75)	0.67***	(0.54, 0.82)
Divorced/separated/widowed	0.71**	(0.57, 0.88)	0.77*	(0.62, 0.95)
Other	2.5	(0.60, 10.35)	2.77	(0.65, 11.72)
**Caregiver HIV status**				
Negative	1.0		1.0	
Positive	1.34***	(1.19, 1.50)	1.29***	(1.15, 1.45)
Undisclosed	0.77**	(0.69, 0.90)	0.76***	(0.66, 0.86)
**Caregiver education level**				
Never attended	1.0		1.0	
Primary incomplete	1.54***	(1.28, 1.86)	1.53***	(1.26, 1.84)
Primary complete	1.60***	(1.42, 1.79)	1.52***	(1.35, 1.71)
Post-primary	2.06***	(1.56, 2.70)	1.93***	(1.47, 2.55)
**Household SES**				
Low	1.0		1.0	
Medium	0.89	(0.78, 1.01)	0.88	(0.78, 1.01)
High	0.73***	(0.64, 0.82)	0.76***	(0.67, 0.86)

***p < 0.001, **p < 0.01, *p < 0.05

OVC age was significantly associated with their linkage to care. Older OVC were more likely to be linked to care than 0–5 years old; 6–10 years OR = 1.39 (95% CI 1.21, 1,59), 11–15 years OR = 1.39 (95% CI 1.22, 1.59), above 15 years OR = 1.45 (95% CI 1.24, 1.68). OVC of disabled caregivers were 24% less likely to be linked to care (OR = 0.76; 95% CI 0.58, 0.99) compared to OVC of other caregivers. OVC of caregivers who were married/cohabiting or divorced/separated/widowed had lower odds of being linked to care, compared to OVC of caregivers who had never been married (OR = 0.67; 95% CI 0.54, 0.82 and OR = 0.77; 95% CI 0.62, 0.95 respectively). OVC of HIV positive caregivers were more likely to be linked to care (OR = 1.29; 95% CI 1.15, 1.45) while OVC of caregivers with undisclosed status were less likely to be linked to care (OR = 0.76; 95% CI 0.66,0.86) compared to OVC of uninfected caregivers. OVC of caregivers who had any education were more likely to be linked to care, compared to those of caregivers who had never attended any school. OVC living in households of high SES were significantly less likely to be linked to care than those living in low household SES (OR = 0.76; 95% CI 0.67, 0.86).

## Discussion

This study assessed how caregivers’ disability and other socio-demographic characteristics are associated with OVC’s linkage to care. Characteristics of caregivers that were significantly associated with OVC linkage to care were disability, marital status, HIV status, education level and household SES. OVC age was also associated with linkage to care.

The findings revealed that OVC of disabled caregivers were 24% less likely to be linked to care, than their counterparts. While there is little literature available on how disabilities of caregivers alter their capacity to look after the health needs of children, the World Health Organization (WHO) reports that globally, people with disabilities have poorer health outcomes than those without [24]. Challenges faced by people with disabilities in accessing healthcare have been widely reported; difficulties in arriving to health facilities, obtaining doctor’s appointments, being attended to in a facility, paying for treatment [25]. A study from rural South Africa reported transport-related issues as a prominent barrier in accessing health facilities [26]. The study also revealed increased barriers for older people with disabilities and those with lower levels of education [26]. Challenges in regards to health care support for OVC have also been widely reported, with caregivers stating inadequate quality of health care, lack of health workers, long distance to the health facilities, financial burden of caring for orphans [27], paying for drugs during high inflation or lack of employment. A study done during a different time period of the Kizazi Kipya project indicated that the highest proportion of OVC lived in households with lowest SES [28], suggesting that economic constraints could be a major player in linkage to care. Interventions that focus solely on the needs of orphans may also not attract caregivers as the benefits would not be shared by other household members [15]. For this study, a combination of the factors identified could be attributed to lower linkage to care for OVC of disabled caregivers. HIV-related stigma is commonly reported [29], as is stigma related to disability [21], therefore disabled caregivers dealing with HIV infection of their OVC face double stigmatization, possibly leading to lower linkage to care [30].

OVC of HIV positive caregivers were more likely to be linked to care. Households with HIV positive caregivers have often been associated with negative child outcomes, such as health adversities, lower school enrollment, poor mental health, exposure to abuse and adolescent risk behavior, consequently increasing risk of HIV [31]. Previous studies have also reported the burden of caregiving for HIV positive children, including food insecurities, difficulty in access to healthcare and economic instability [32]. To overcome these adverse outcomes, community-based care models have been encouraged [32, 33]. To this end, the finding that linkage to care is higher among OVC living with HIV positive caregivers indicates a strength of implementing the Kizazi Kipya program, in terms of making services accessible to the double burdened households.

The study found higher likelihood of linkage to care among OVC of caregivers with higher education level. A qualitative study had reported a likelihood of hampered access to health information and adherence to ART for caregivers with low education level [32]. Another study reported low HIV knowledge among caregivers with low level of education, associated with lower disclosure of their child’s HIV status [34]. Similarly, OVC of caregivers with higher education in this study reported being linked to care. This could be attributed to higher HIV knowledge among caregivers with higher education and their willingness to accept care for their OVC. This is an important finding for the project, as community workers who serve households of caregivers with low literacy level should receive additional support in terms of creating awareness around the importance of linking OVC to care and provide referrals for the same.

Interestingly, OVC living in households of higher SES were less likely to be linked to care than those with lower SES. While reasons behind this would have to be understood through further analysis of household level characteristics and qualitative study, one of the reason could be that caregivers of OVC with higher household SES prefer and are able to seek care at private facilities. Using multi-country Demographic Health Survey (DHS) data, a study that assessed utilization of private health sector for HIV-related services showed positive association between income and use of private health facilities for sexually transmitted infections (STIs) care [35]. Another reason for not reporting to be linked to care could be HIV-related stigma. However, previously done studies have reported mixed findings; with increased HIV prevalence, poorer HIV outcomes [36] and increased HIV related stigma [29] among individuals in lower and middle SES, while some evidence also showed higher HIV prevalence among wealthier individuals in sub-Saharan African countries, associated to increased risky behaviors and having multiple sexual partners [37].

Also, linkage was more likely as OVC grew older, suggesting the possibility of increased self-initiatives for self-care as age advances. This may come as an addition to the readily available caregiver support and consequently magnify the OVC’s likelihood to be linked to care and treatment as their age exceeds 5 years. Previous studies have also reported other treatment support provided to school-aged children, such as appointment reminders and treatment buddies [38], to be effective in linkage and retention to care among older children. Therefore, the younger OVC, especially those in the youngest age group (0–5 years) may require more tailor-made support as their linkage may entirely depend on the external environment.

OVC of caregivers who had ever been married were less likely to be linked to care than OVC of caregivers who had never been married. Although there is not enough literature to support this, it could be because unmarried caregivers would have fewer responsibilities in the household, compared to ever married caregivers who would be looking after their spouse, children or in-laws, therefore being more able to commit to accessing HIV facilities with their OVC. Unmarried caregivers might also be younger (siblings or cousins of OVC) than ever married caregivers (grandparents of OVC), who would be better able to support their OVC.

### Strengths and limitations

A major strength of this study is the wide geographical coverage, ensuring that the results are applicable to whole of Tanzania. There is a concern that the future of HIV epidemic and its effects lies within small segments of the unreached population, such as the disabled people. To the authors’ knowledge, this is one of the few studies in Tanzania that focuses on the association between caregiver disability and HIV care for the OVC in their households. The authors believe this study provides a much needed insight and opens possibilities for future studies. Although the proportion of disabled caregivers was only 3% in this study, it is sufficient given the study focused on disabled caregivers who were majorly affected by HIV and were taking care of OVCLHIV, given the low reported proportion of disabled adults in the country overall. However, the study findings do not tell more about the mechanisms through which caregivers’ disability hampers OVC linkage to care, rather, quantify the commonly reported barriers. Further qualitative studies would be needed to understand these mechanisms. Factors such as distance to the facility, individual perceptions on need for services or stigma associated with seeking HIV care, severity of caregivers’ disability, which could influence OVC’s linkage to care, were also not available for this study.

## Conclusion

The findings from this study indicate a need for implementing programs and making policies that focus on the needs of disabled individuals and their household members, especially those affected by HIV. While a small proportion of the caregivers in this study were disabled, the challenges generally reported in literature are highlighted in the results. The findings also add to the limited existing knowledge on how disability of caregivers can be associated with children’s access to essential services. The findings can be used to improve the existing project activities, by focusing on disability led households, as well as include disability as a main eligibility criterion during screening and enrollment of participants in future large-scale programs.

## Data Availability

This study is based on data from the USAID Kizazi Kipya project (2016–2021) in Tanzania. Pact Tanzania is the primary organization implementing the project, hence owns the data. The datasets analyzed during the current study are not publicly available due to confidentiality restrictions, but are available from Pact, Tanzania on a reasonable request.

## Abbreviations

ART: Anti-retroviral therapy
CI: Confidence interval
CTC: Care and treatment clinics
CW: Community workers
DHS: Demographic Health Survey
FCAA: Family and Child Asset Assessment
GEE: Generalized estimating eqs.
HIV: Human immune deficiency virus
OR: Odds ratios
OVC: Orphans and vulnerable children
OVCLHIV: Orphans and vulnerable children living with HIV
PCA: Principle component analysis
PLHIV: People living with HIV
SES: Socio-economic status
UNAIDS: The Joint United Nations Programme on HIV/AIDS
WHO: World Health Organization
